# Vitamin D Status Is Positively Correlated with Regulatory T Cell Function in Patients with Multiple Sclerosis

**DOI:** 10.1371/journal.pone.0006635

**Published:** 2009-08-13

**Authors:** Joost Smolders, Mariëlle Thewissen, Evelyn Peelen, Paul Menheere, Jan Willem Cohen Tervaert, Jan Damoiseaux, Raymond Hupperts

**Affiliations:** 1 School for Mental Health and Neuroscience, Maastricht University Medical Center, Maastricht, The Netherlands; 2 Department of Internal Medicine, Division of Clinical and Experimental Immunology, Maastricht University Medical Center, Maastricht, The Netherlands; 3 Department of Clinical Chemistry, Maastricht University Medical Center, Maastricht, The Netherlands; 4 Department of Neurology, Orbis Medical Center, Sittard, The Netherlands; New York University School of Medicine, United States of America

## Abstract

**Background:**

In several autoimmune diseases, including multiple sclerosis (MS), a compromised regulatory T cell (Treg) function is believed to be critically involved in the disease process. *In vitro*, the biologically active metabolite of vitamin D has been shown to promote Treg development. A poor vitamin D status has been linked with MS incidence and MS disease activity. In the present study, we assess a potential *in vivo* correlation between vitamin D status and Treg function in relapsing remitting MS (RRMS) patients.

**Methodology/Principal Findings:**

Serum levels of 25-hydroxyvitamin D (25(OH)D) were measured in 29 RRMS patients. The number of circulating Tregs was assessed by flow-cytometry, and their functionality was tested *in vitro* in a CFSE-based proliferation suppression assay. Additionally, the intracellular cytokine profile of T helper cells was determined directly ex-vivo by flow-cytometry. Serum levels of 25(OH)D correlated positively with the ability of Tregs to suppress T cell proliferation (R = 0.590, P = 0.002). No correlation between 25(OH)D levels and the number of Tregs was found. The IFN-γ/IL-4 ratio (Th1/Th2-balance) was more directed towards IL-4 in patients with favourable 25(OH)D levels (R = −0.435, P = 0.023).

**Conclusions/Significance:**

These results show an association of high 25(OH)D levels with an improved Treg function, and with skewing of the Th1/Th2 balance towards Th2. These findings suggest that vitamin D is an important promoter of T cell regulation *in vivo* in MS patients. It is tempting to speculate that our results may not only hold for MS, but also for other autoimmune diseases. Future intervention studies will show whether modulation of vitamin D status results in modulation of the T cell response and subsequent amelioration of disease activity.

## Introduction

Multiple Sclerosis (MS) is a disabling, chronic inflammatory disease of the central nervous system (CNS). Although some patients experience progression of disability from the first symptoms onwards, the disease is at its onset mostly characterised by repetitive sub-acute deteriorations of symptoms with remissions afterwards, i.e. relapsing remitting MS (RRMS). After several years, sustained and progressive disability often occurs and the disease becomes secondary progressive [1].

Although the pathophysiological process in MS is not yet fully elucidated, T cell mediated inflammation within the CNS plays an important role. Auto-reactive T cells move through the blood brain barrier, and contribute to local inflammation of the CNS [2]. Historically, these auto-reactive T cells were believed to comprise predominantly interferon gamma (IFN-γ) producing T helper 1 (Th1) cells. More recently interleukin-17 (IL-17) producing Th17 cells have been argued to be important pathogenic T cells in MS [3], [4]. In a healthy T cell compartment, regulatory T cells (Treg) regulate the quantity and quality of the immune response, and prevent auto-reactive T cell responses, and hereby autoimmune diseases [5]. Although the number of Tregs is not decreased, their capability to suppress polyclonal or antigen-specific proliferation of T cells *in vitro* has been shown to be compromised in MS patients [6]–[8].

Experimental studies showed that the biologically active metabolite of vitamin D, 1,25-dihydroxyvitamin D (1,25(OH)_2_D), is able to skew the T cell compartment into a more anti-inflammatory and regulated state, with inhibition of Th1 and Th17 cells and promotion of Th2 and Treg cells [9], [10]. Interestingly, in the experimental autoimmune encephalomyelitis (EAE) animal model of MS, treatment with vitamin D prevented, and even cured, the disease [11]. These findings suggest a potential role for vitamin D in MS, as has been investigated in several epidemiological studies. Near the equator, where vitamin D photosynthesis is optimal, MS incidence is low [12]. Furthermore, increased sun exposition and a good vitamin D status in childhood and adolescence, reflected by the serum values of 25-hydroxyvitamin D (25(OH)D) [13], have been associated with a decreased risk for developing MS [14]–[16]. An increased activity and disability of MS has also been associated with a poor vitamin D status [17]–[19]. Since vitamin D modulates the immune response *in vitro* and in animal models, it is highly interesting to assess whether this modulation can also be found in vivo in MS patients. This could be, beside regulation of the transcription of MS-associated alleles [20], an important driver of the associations between vitamin D status and MS. At present, however, no published studies assessed the potential relationship between serum levels of 25(OH)D and the T cell compartment in MS.

In the present study, we assess whether vitamin D status is correlated with the *in vivo* composition and function of the T cell compartment in MS patients. Since in RRMS patients with a short disease duration disease pathophysiology has been argued to be predominantly inflammatory [1] and Treg function has been shown to be particularly compromised [7], we assess a potential anti-inflammatory and in particular Treg-promoting effect of vitamin D, as being related to vitamin D status, in these patients.

## Materials and Methods

### Subjects

We included in this study 29 Caucasian subjects, by approaching consecutive patients matching the inclusion criteria, who visited the outpatient clinic of our MS centre. All subjects had RRMS according to the revised McDonald criteria [21], with a disease duration of <5 years since the first to MS attributable symptoms. Both patients treated with interferon beta 1a or 1b, and patients untreated with immune modulation were included. Exclusion criteria were: no clinically definite phenotype, use of other immune modulation than interferon beta 1a or 1b, occurrence of a relapse <6 weeks before blood collection, recent start (<^1^/_2_ year) of vitamin D supplementation. The characteristics of the patients are described in Table 1.

**Table 1:** Patient characteristics.

	Median/N (%)	Range [min–max]
Male/Female (N)	10 (34%)/19 (66%)	
Age (years)	39.23	[22.61–58.63]
Duration MS (years)	3.48	[0.69–4.89]
Relapse Rate (N/year)	0.00	[0.00–3.00]
Relapse free (years)	1.04	[0.10–3.91]
EDSS* score (points)	2.00	[0.00–6.00]
Interferon beta treated (N)	25 (86%)	

* Expanded Disability Status Scale.

### Ethical statement

Written informed consent was acquired from all subjects participating in this study, according to the declaration of Helsinki. The study was reviewed and approved by both the regional ethical committee on human research ‘Atrium-Orbis-Zuyd’, and the institutional ethical committee ‘Local Advisory board on Scientific Research’.

### Vitamin D assays

Blood withdrawal was performed in the period from September 2008 until February 2009. The cells for the cellular assays and the serum for vitamin D measurement were retrieved at the same time, and cellular assays were performed on the day of blood collection. The collected serum was immediately shielded from direct light and stored at −20°C. At the end of the study, all samples were analysed simultaneously. The serum values of 25(OH)D were measured with a commercially available radioimmuno assay (Immunodiagnostic Systems, Boldon, UK) [18].

### Cell culture reagents

Cells were cultured in RPMI glutamax medium (Gibco Invitrogen, Breda, The Netherlands) supplemented with 10% Foetal Calf Serum (Greiner Bio-One, Alphen a/d Rijn, The Netherlands), 1% non-essential amino acids (Gibco Invitrogen), 1% sodium pyruvate (Gibco Invitrogen) and 2% penicillin-streptomycin (Gibco Invitrogen) in U-bottom 96-wells plates.

### Cell purification

**Figure 1:**
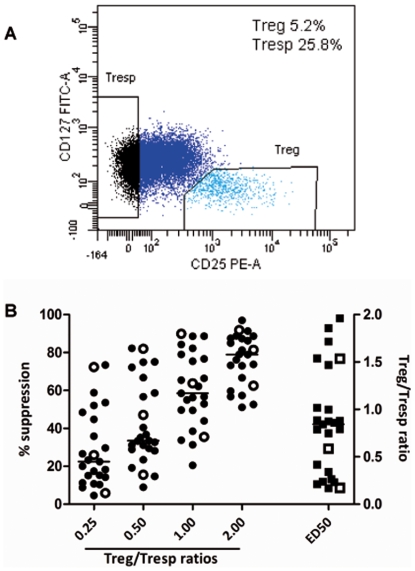
Results proliferation suppression assay. (A) Representative dot-plot of the sorting protocol. CD25^+^CD127^−^ cells within the CD4^+^ lymphogate were sorted as Tregs, CD25^−^ cells were sorted as Tresps. (B) The percentages of suppression which were achieved for the respective Treg/Tresp ratios of the individual patients are shown. The Treg/Tresp ratio at which 50% inhibition of proliferation was achieved is defined as ED50 of suppression. The distribution of ED50 is shown in the rightmost column. The lines show the median values. The closed dots represent the Beta Interferon-treated patients, the open dots the untreated patients.

Cell isolation was performed as described before, with minor modifications [22]. PBMC were isolated by Ficoll gradient centrifugation (Histopaque; Sigma Aldrich, Zwijndrecht, The Netherlands). CD4^+^ T cells were selectively isolated with RosetteSep (Stem Cell Technologies, Grenoble, France). Approximately 20–30×10^6^ CD4^+^ T cells were incubated at 4°C for 30 minutes with anti-CD4-APC (BD Biosciences, Breda, The Netherlands), anti-CD25-PE (BD Biosciences) and anti-CD127-FITC (BD Biosciences). Human CD4^+^CD25^+^CD127^−^ Tregs [23], [24] and CD4^+^CD25^−^ responder T cells (Tresps) were sorted on a FacsAria^TM^ (BD Biosciences) cell sorter (Figure 1A). The mean purity of the sorted populations was 97.51% (SD 0.81) for the Tregs and 97.45% (SD 1.65) for the Tresps. Accessory cells were obtained by irradiating autologous PBMC with 66.2 gray.

### Proliferation assay

The CFSE-based proliferation suppression assay was adapted from Venken et al. [22]. In short, the freshly isolated CD4^+^CD25^−^ Tresps were labelled with carboxy fluorescein diacetate succinimidyl ester (CFSE; Molecular Probes Invitrogen, Breda, The Netherlands). In a U-bottom 96-wells plate, 2×10^5^ Tresps were stimulated with soluble anti-CD3 (2.0 pg/mL, WT32 IgG2a monoclonal antibody, kindly provided by dr. W Tax, Radboud University Nijmegen Medical Centre, Nijmegen, The Netherlands) in the presence of 2×10^5^ irradiated accessory cells. Tresps were co-cultured for 5 days with varying amounts of Tregs (Treg/Tresp ratios 0/1, 0.25/1, 0.5/1, 1/1 or 2/1). All conditions were performed in triplo in a final volume of 200 µL. After culture, the cells were stained with anti-CD4-APC and 7AAD (BD Biosciences), and the CFSE signal of cells in the CD4^+^7AAD^−^ lymphogate was analysed by Flow Cytometry on a FacsCalibur^TM^ flowcytometer (BD Biosciences). Data were analysed with CellQuest software (BD Biosciences). The amount of proliferation achieved in the 0/1 ratio was set at 0% suppression. The mean relative amount of suppression in the other ratios was calculated. By linear interpolation, the Treg/Tresp ratio at which 50% suppression of proliferation was achieved (ED50) was calculated.

### T cell phenotyping

Tregs were defined as CD25^+^FoxP3^+^ CD4^+^ T cells [25], [26] and as CD25^+^CD127^−^ CD4^+^T cells [23], [24]. For determining the proportion of Tregs, PBMC were stained with anti-CD3-PerCP (BD Biosciences), anti-CD4-APC, anti-CD4-FITC (BD Biosciences), anti-CD25-PE, anti-CD127-FITC or anti-FoxP3-APC (E-Bioscience, San Diego, USA), and analysed on a FacsCalibur^TM^ flowcytometer [27]. The absolute number of lymphocytes was determined with a haematological cell counter (Beckman Coulter, Woerden, The Netherlands).

T helper cell subsets were determined by assessing the intracellular cytokine pattern of CD3^+^CD8^−^ lymphocytes, which are further referred to as CD4^+^ T cells, by flowcytometry [27]. PBMC were stimulated for 5 hours with calcium ionomycine (Sigma Aldrich), phorbol 12-myristate 13-acetate (PMA; Sigma Aldrich) and monensin (BD Biosciences). Cells were stained intra-cellularly with anti-IL-4-PE (BD Biosciences), anti-IFN-γ-FITC (BD Biosciences), anti-IL-17-PE (E-Bioscience) and anti-IL-10-PE (E-Bioscience), and extra-cellularly with anti-CD3-PerCP and anti-CD8-APC.

### Statistics

Statistical analysis was conducted with Statistical Package for Social Sciences version 15.0 software (SPSS inc., Chicago IL, USA) and figures were constructed with GraphPad Prism 5 software (GraphPad Software inc., La Jolla CA, USA). Of continuous variables, the median value and corresponding range (min–max) are provided. When normally distributed (Shapiro-Wilk test P>0.05), a linear relationship between two continuous variables was tested with the Pearson correlation coefficient (Pearson R). Abnormally distributed variables were Log-transformed. In case of persistent abnormal distribution, the Spearman correlation coefficient (Spearman R) was determined. A P value<0.05 was considered statistically significant.

## Results

### Serum 25(OH)D levels

We determined the serum levels of 25(OH)D, the vitamin D metabolite which is accepted to reflect vitamin D status best [13]. The median serum 25(OH)D level was 54 nmol/L (19–133).

### Suppressive capacity of Tregs correlates positively with 25(OH)D levels

**Figure 2:**
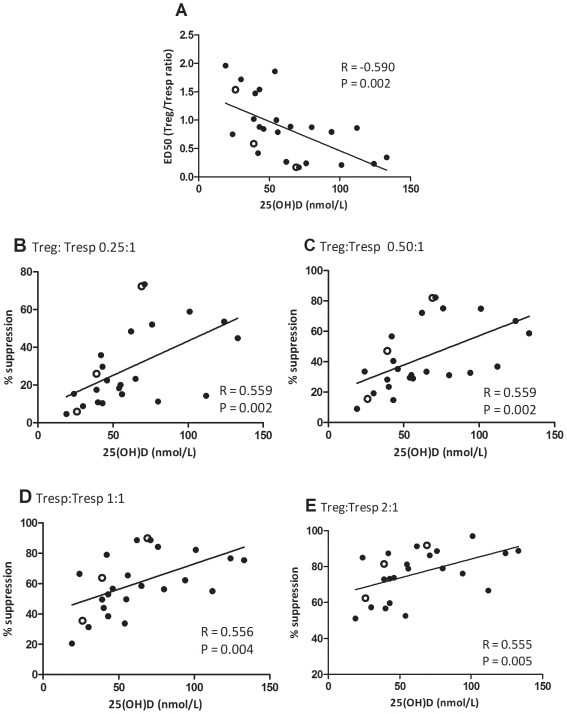
Correlation between Treg suppressive function and serum 25(OH)D levels. (A) The correlation between ED50 and serum 25(OH)D levels. (B–E) Correlation between inhibition of suppression and 25(OH)D levels for the individual Treg/Tresp ratios. The Spearman correlation coefficient is shown. The regression line illustrates the direction of the association. The closed dots represent the Beta Interferon-treated patients, the open dots the untreated patients.

To evaluate Treg suppressive capacity, we conducted an in vitro CFSE-based proliferation suppression assay (Figure 1B). Of 4 patients, not enough cells were retrieved to start the assay. In all other individual patients, Tregs suppressed proliferation of T responder cells in a dose-dependent manner. There was no apparent difference in Treg suppression between Beta Interferon treated and untreated patients (Figure 1B). The median Treg/Tresp ratio at 50% suppression of proliferation (ED50) was 0.82 (0.16 - 1.96). Serum 25(OH)D levels correlated negatively with ED50 (Spearman R = −0.590, P = 0.002) (Figure 2A). This correlation was reflected by the positive correlation between vitamin D status and the amount of suppression in the individual Treg/Tresp ratios (Figure 2B–E).

### Number of circulating Tregs does not correlate with 25(OH)D levels

**Figure 3:**
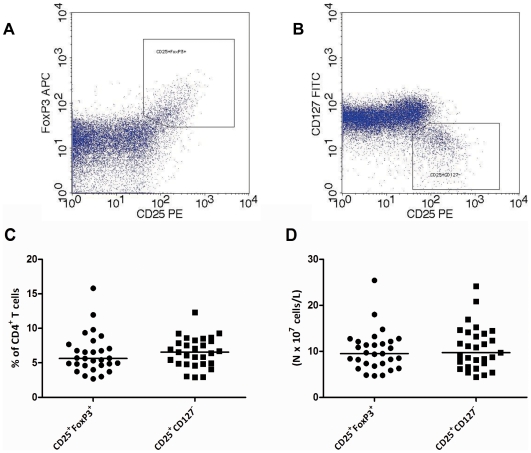
Relative and absolute number of circulating Tregs. Within the CD3^+^CD4^+^ lymphogate, Tregs were defined as either being (A) CD25^+^FoxP3^+^ or being (B) CD25^+^CD127^−^. (C) The percentages of Tregs within the CD4^+^ T cell compartment. (D) Absolute number of circulating Tregs. The lines indicate the median values.

**Figure 4:**
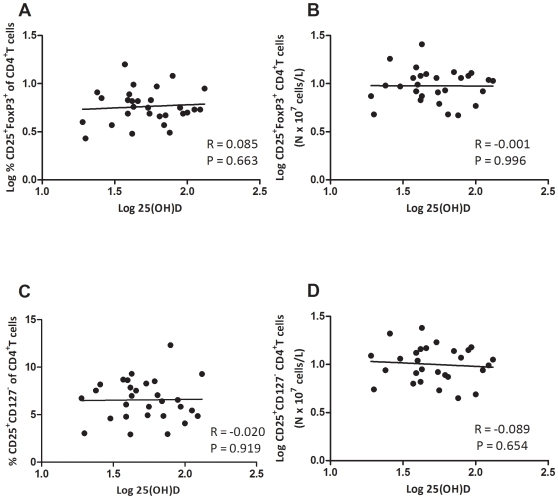
Correlation of the number of circulating Tregs with serum 25(OH)D levels. (A–D) Correlation of relative and absolute number of Tregs with 25(OH)D levels. The Pearson correlation coefficient and corresponding regression line are shown.

Next, we evaluated the absolute and relative number of Tregs in the circulation (Figure 3). Regulatory cells within the CD4^+^ T cell compartment were defined as CD25^+^CD127^−^ and CD25^+^FoxP3^+^ cells. The median percentage of CD25+FoxP3+ Tregs in the CD4+ T cell compartment was 5.63% (2.67–15.81) (absolute number 9.61×107 c/L (4.65–25.41)). The median percentage of CD25+CD127− Tregs was 6.56% (2.93–12.32) (absolute 10.32×107 c/L (4.44–24.14)). A strong positive correlation was observed between the numbers of CD25^+^CD27^−^ and CD25^+^FoxP3^+^ CD4^+^Tregs (R = 0.826, P<0.001). Serum levels of 25(OH)D were not correlated with the relative and absolute number of Tregs (Figure 4).

### The Th1/Th2 balance correlates negatively with 25(OH)D levels

**Figure 5:**
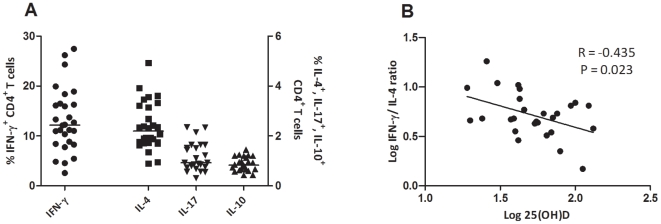
Correlation of T helper cell subsets with serum 25(OH)D levels. (A) The percentages of IFN-γ^+^, IL-4^+^, IL-17^+^ and IL-10^+^ CD4^+^ T cells. The lines show the median values. (B) The correlation between IFN-γ/IL-4 ratio and serum 25(OH)D levels. The Pearson correlation coefficient and corresponding regression line are shown.

CD4^+^ T cells were classified as Th1, Th2 or Th17 by assessing their intracellular cytokine profile (IFN- γ, IL-4 and IL-17, resp.) (Figure 5A). We also assessed the percentage of CD4^+^ T cells producing the regulatory cytokine IL-10 [9]. The median percentage of IFN-γ^+^ cells within the CD4^+^ T cell compartment was 12.11% (2.60 - 26.18), of IL-4^+^ cells 2.07% (0.90–4.93), of IL-17^+^ cells 0.92% (0.31–2.35), and of IL-10^+^ cells 0.83% (0.43–1.45). No significant correlations between serum 25(OH)D levels and the individual percentages of T helper cell subsets were found, although there was a trend towards a negative correlation with the percentage of IFN-γ^+^CD4^+^ T cells (Pearson R = −0.336 P = 0.060). The balance between Th1 and Th2 is used to indicate a more pro- or anti-inflammatory composition of the T cell compartment [4]. This balance is described by the ratio of IFN-γ^+^ and IL-4^+^ CD4^+^ T cells. The IFN-γ/IL-4 ratio correlated negatively with serum 25(OH)D levels (Pearson R = −0.435 P = 0.023) (Figure 5B).

### No association between MS disease activity and vitamin D status or T cell parameters

Neither vitamin D status, nor T cell parameters as Treg function or T cell phenotype did correlate with measures of MS disease activity as described in table 1 (data not shown).

## Discussion

Vitamin D status has been linked with MS either in large epidemiological studies or in experimental *in vitro* and animal studies. This is the first study to investigate the correlation between vitamin D status and the composition of the T cell compartment and in particular Treg function *in vivo* in MS patients. Our data shows that serum 25(OH)D levels correlate positively with the ability of CD4^+^CD25^+^CD127^−^ Tregs to suppress proliferation of Tresp cells. The absolute and relative Treg numbers in the circulation were not correlated with vitamin D status. Although we did not observe a significant association between 25(OH)D levels and the individual percentages of CD4^+^ T helper cell subsets, the decreasing IFN-γ/IL-4 ratio reflected a skewing of the Th1/Th2-balance towards a more Th2 phenotype in patients with higher serum 25(OH)D levels. Altogether, this data indicates that high serum 25(OH)D levels are associated with a less inflammatory and better regulated T cell compartment in MS.

In our data, we found no association between vitamin D status and disease severity and activity of MS. This is in contrast with earlier observations [17]–[19]. However, since we measured patients in the period from September until February, the seasonal fluctuation of vitamin D status is likely to confound these associations [28]. In contrast to the MS activity variables, this seems not critical for the correlation with the T cell tests: the data obtained reflects the status of the T cell compartment at the moment of blood collection. Hereby, several studies have shown a seasonal fluctuation of immunological parameters in MS patients comparable to the fluctuation of serum 25(OH)D levels [29], [30].

In a previous study, CD4^+^CD25^high^ Tregs of RRMS patients achieved only about 40% suppression in a 1∶1 Treg/Tresp ratio as measured in a ^3^H-Thymidine assay [8]. A later study confirmed a mean suppression of 40% in untreated RRMS patients, and of 50% in Beta interferon users [7]. Our RRMS patients reached in the 1∶1 ratio a mean suppression of 60% (20–90). Beta Interferon treatment has been shown to promote Treg function [31]. Therefore, the fact that most of our patients were treated with Beta Interferon might have contributed to this substantially more favourable suppression. Additionally, we included the α-chain of the IL-7 receptor (CD127), which has been shown to be specifically absent on CD4^+^CD25^+^FoxP3^+^ Tregs, in our sorting protocol to define Tregs [23], [24]. Therefore, the sorted population of CD4^+^CD25^+^CD127^−^ Tregs may comprise a more pure Treg fraction, compared to CD4^+^CD25^high^ sorted T cells alone [32].

Our data shows that in RRMS patients with a short disease duration, in which Treg function has been shown to be particularly compromised [7], Treg suppressor function correlates positively with serum 25(OH)D levels. The relationship between Treg function and 25(OH)D levels is likely to contribute to the inter-individual variation of Treg suppressive function in MS cohorts in other studies [6]–[8]. However, we could not find an association of serum vitamin D values with the number of CD4^+^CD25^+^FoxP3^+^ or CD4^+^CD25^+^CD127^−^ Tregs in the circulation. This discrepancy between the impaired number and function of Tregs has been shown in other studies on Tregs in MS and other autoimmune diseases [33]. However, the underlying mechanism is not know. Interestingly, a recent publication showed a negative correlation between the percentage of FoxP3^+^ Tregs within the T cell compartment of MS patients and their vitamin D status [34]. However, the heterogeneous study population considering race and disease duration, and the use of linear regression models to analyse non-equally distributed parameters in a limited sample size, require careful interpretation of these results. The discrepancy with our data not showing a comparable correlation, might be attributable to these methodological differences.

Additionally, we found a negative correlation between serum 25(OH)D levels and the IFN-γ/IL-4 ratio. This ratio is commonly used to describe the balance in the immune system between pro-inflammatory IFN-γ^+^ Th1 cells and anti-inflammatory IL-4^+^ Th2 cells [4]. Our data suggests that, in patients with relatively high 25(OH)D-levels, the balance between Th1 and Th2 is more skewed in the direction of Th2. Interestingly, *in vitro* research also suggested that vitamin D skews the T cell compartment from a Th1 towards a Th2 phenotype [9], [10]. Therefore, high 25(OH)D D levels appear to be associated with a less-pro-inflammatory T-cell compartment in MS patients.

In conclusion, we have shown that serum 25(OH)D levels are associated with the suppressive function of Tregs and the composition of the T cell compartment in MS patients. These findings further support the hypothesis that vitamin D is an important promoter of T cell regulation in MS. Since in RRMS patients the Treg compartment has been shown to be compromised, our findings provide additional arguments to maintain a healthy vitamin D status in these patients. Also other autoimmune diseases have been associated with both low vitamin D levels, and a reduced regulatory T cell function [33], [35]. Whether a poor vitamin D status correlates in healthy populations with a reduced Treg function and potentially a subsequently increased risk for developing autoimmune diseases, remains to be established. Altogether, it is tempting to speculate about the potential therapeutic effect of vitamin D supplementation in autoimmune diseases. Previously, it was demonstrated that therapy with Beta Interferon in MS results in a promotion of regulatory T cell function [31] and a decrease of relapse rate [36]. Since we observed an additional effect of 25(OH)D levels in Beta Interferon users, this might provide a rationale for add-on therapy with vitamin D during Beta Interferon therapy in MS. Randomized clinical trials should assess an additional role of vitamin D treatment on the T cell compartment and more importantly on disease activity of MS.
